# Impact of a motivational resistance-training programme on adherence and body composition in the elderly

**DOI:** 10.1038/s41598-018-19764-6

**Published:** 2018-01-22

**Authors:** Pablo Jorge Marcos-Pardo, Alejandro Martínez-Rodríguez, Alexander Gil-Arias

**Affiliations:** 1 Faculty of Sports, Catholic University San Antonio of Murcia (UCAM), Murcia, Spain; 2 Faculty of Science, University of Alicante, Alicante, Spain; 3 Sports Studies Center, Rey Juan Carlos University, Alcorcón (Madrid), Spain; 4 Present Address: Faculty of Sports, Catholic University San Antonio of Murcia (UCAM), Av. de los Jerónimos, 135, 30107 Murcia, Spain

## Abstract

Lack of physical activity is one of the major causes for obesity and functional disability in the elderly. Including regular exercise in the elderly’s lifestyle is not an easy task. The main objective was to analyse the effect of a motivational resistance-training programme on satisfying the individual’s psychological needs, level of self-determination and body composition. A quasi-experimental study was performed with 47 volunteers (29 females, 18 males) of 67–75 years of age, divided into two groups: experimental (n = 27) and control (n = 20). A 12-week intervention programme was performed, with a total of 36 sessions. The results of the inter-group analysis indicated significant differences in the post-test measurement between the experimental group and the control group (in favour of the experimental group) regarding basic psychological needs. The experimental group, in comparison to the control group, significantly decreased their percentage of fat mass and increased muscle mass. Body weight and BMI values increased in the control group, while significantly decreasing in the experimental group. In conclusion, the motivational resistance-training programme in the elderly gave rise to positive significant changes at the physical, psychological and social levels, according to the definition of health by the World Health Organization.

## Introduction

The level of physical activity in the older population decreases with age. This not only affects the intensity of the exercise, but also its duration, which ultimately results in difficulties in performing daily tasks and overall decreased quality of life^1,2^. This state of physical inactivity is considered to be one of the major causes of obesity, mortality and functional disability in the aging population^3–5^. Therefore, a proper strategy to increase life expectancy and maintain the individual’s wellbeing is to exercise regularly, even at advanced age^6,7^. Even though aging should not be regarded as a terminal state, it does cause structural and functional changes, which progressively affect an individual’s capabilities, and consequently, their health and quality of life^1,8–13^.

The aging process is associated with a progressive loss of muscle mass, strength, potency and muscle resistance^14–18^. This progressive loss of muscle mass and function, known as sarcopenia, results in frailty, comorbidities and mortality and affects 30% of the older population (over 65 years of age)^19,20^.

For decades, research has shown that exercise and caloric restriction can postpone detrimental aspects of aging^5,21^. Despite all the evidence for the positive role of regular physical activity and exercise in long-term health management, it remains unclear why such a low percentage of individuals seeking a healthy lifestyle are capable of successfully integrating physical activity behaviours into their lifestyles and achieving long-lasting improvements in fitness^22^. Physical exercise is an effective approach in promoting health^3,23–25^, wherein the physical conditioning of those who regularly participate in physical activities increases and/or improves both their physical and psychological capacities^26,27^.

Resistance-training improves muscle strength and contributes to maintaining functional autonomy in the elderly^10,28^. At the same time, resistance-training in older adults also increases strength, enhances energy expenditure, reduces the difficulty of performing daily tasks and induces changes in body composition^29–32^. In the short term, one of the best investments older people can make to live longer, delay aging, and enjoy healthier lives are regular physical aerobic and muscle-strengthening activities^33^.

In this sense, various studies have shown that resistance-training in the elderly can induce changes in body composition, decreased fat mass and increased muscle mass being the most relevant^12,31^. Similarly, experimental studies have shown that individuals performing resistance-training significantly reduce fat-free mass^9^. Therefore, performance of resistance-training could be an indicator of a healthy body composition^12,31^.

The inclusion of exercise as a consistent lifestyle behaviour is not an easy task for many elders due to low exercise tolerance and enjoyment^34^. Optimal body weight control and reduced central obesity risk may have beneficial effects on health^35^. Several factors can hamper physical activity in obese and normal-weight individuals, such as low motivational status, self-efficacy, negative learning history in relation to exercise, lack of coping skills, and aversive environmental characteristics such as reduced access to physical activity facilities, high costs of training programmes, low social and cultural support, and time barriers^36^. For this reason, new exercise strategies have been suggested that could present higher levels of adherence, which consider both intrinsic and extrinsic factors that may alter the individual’s motivation.

Current theories and studies regarding the psychological predictors of success in exercise suggest that the Self-Determination Theory (SDT)^37,38^ seems to be the best method to explain the types and levels of motivation that are associated with initiation and maintenance of behavioural changes that reflect attitudinal responses. SDT considers that the origin of the motivation may be internal or external (more or less self-determination) as the individual is more or less involved in the performance of their activities^39^. To this end, the theory establishes that motivation is represented by a continuum that discerns 3 types of self-determination, ordered from more to less self-determination: autonomous motivation, controlled motivation and amotivation^40^. Autonomous motivation is comprised of (in descending order regarding level of self-determination) intrinsic motivation, integrated regulation and identified regulation. These types of motivation are evident when the activities are performed for pleasure and leisure, since they are highly interesting activities for the individual (intrinsic regulation) or because they form part of their lifestyle (integrated regulation), or have a high personal value when the individual is conscious of the importance and benefits of physical exercise (identified regulation). Controlled motivation is composed of less self-determining regulations (in descending order): introjected regulation, referring to situations when behaviour is due to internal pressures (i.e. search for self-esteem and avoidance of sense of guilt); and external regulation, which manifests when the behaviour is performed as a consequence of external pressures, such as threats of punishment, rewards or recognition by other individuals^41^. Lastly, amotivation represents the lowest level of self-determination^38^, and refers to the lack of interest of the individual to perform a task, resulting in disorganization and sense of frustration, depression or fear^42^.

The SDT identifies three basic psychological necessities: the necessity for competence, referring to the feeling that an individual may perceive when he/she is efficient in a certain activity; autonomy, refers to the individual following his/her own initiative and having the sensation of choice; and social relations, defined as the feeling of being part of a group and highly regarded by other individuals. These three basic psychological needs (BPN) are essential for an optimal functioning of the natural progress towards growth and integration, as well as social/constructive development and personal wellbeing^38,42^. The SDT establishes that satisfying the BPN of autonomy, competence and social relations gives rise to the development of more self-determining motivations^43–47^. The Hierarchical Model of Motivation (HMM)^48^ was developed in order to improve and associate these BPN with SDT motivational constructs^49^. According to the HMM, sports instructors, through their intervention in the sessions (i.e. providing information and interacting with the participants) greatly contribute to satisfying basic psychological needs, and consequently, support the level of autonomous motivation. This level of self-determined motivation can predict positive or negative cognitive, affective and behavioural outcomes (i.e., intention to be physically active).

In this sense, it is important to note that for the older population to integrate regular physical exercise into their routines, they must not only know the benefits of this practice, but also the sessions must be designed based on their specific needs, as well as the tasks adjusted to their level of competence. Also, they must be performed in a way that social relations are encouraged, so that the motivational component may act as a determining factor in the performance and continuance of the sports practice, with the goal of becoming more physically active^50^.

Despite the importance that motivational variables exert on adherence to physical exercise routines, experimental studies have generally focused on determining the effect training programmes may have on biological variables, such as VO_2_ max^51–53^, potency^33,54,55^, or body composition^56,57^, etc. However, there is no published study that analyses the effect of a multi-dimensional training programme on motivational variables, therefore assessing not only the physical benefits but also the psychological and social ones, which are integrated into the definition of health as defined by the World Health Organization^58^.

Consequently, the main objective of this study was to analyse the effect of a multi-dimensional, moderate-high intensity, resistance-training programme in the aging population, where motivational strategies were included in order to satisfy the participants’ basic psychological needs, as well as improving their level of self-determination and body composition. The following hypotheses were considered from this general objective: (1) the motivational strategies of the training programme must increase the level of satisfaction of the BPN, and consequently, a more self-determining motivation and adherence, with respect to the control group; (2) the training programme will cause changes in their body composition, reducing fat mass and BMI while increasing muscle mass.

## Material and Methods

### Participants

**Figure 1:**
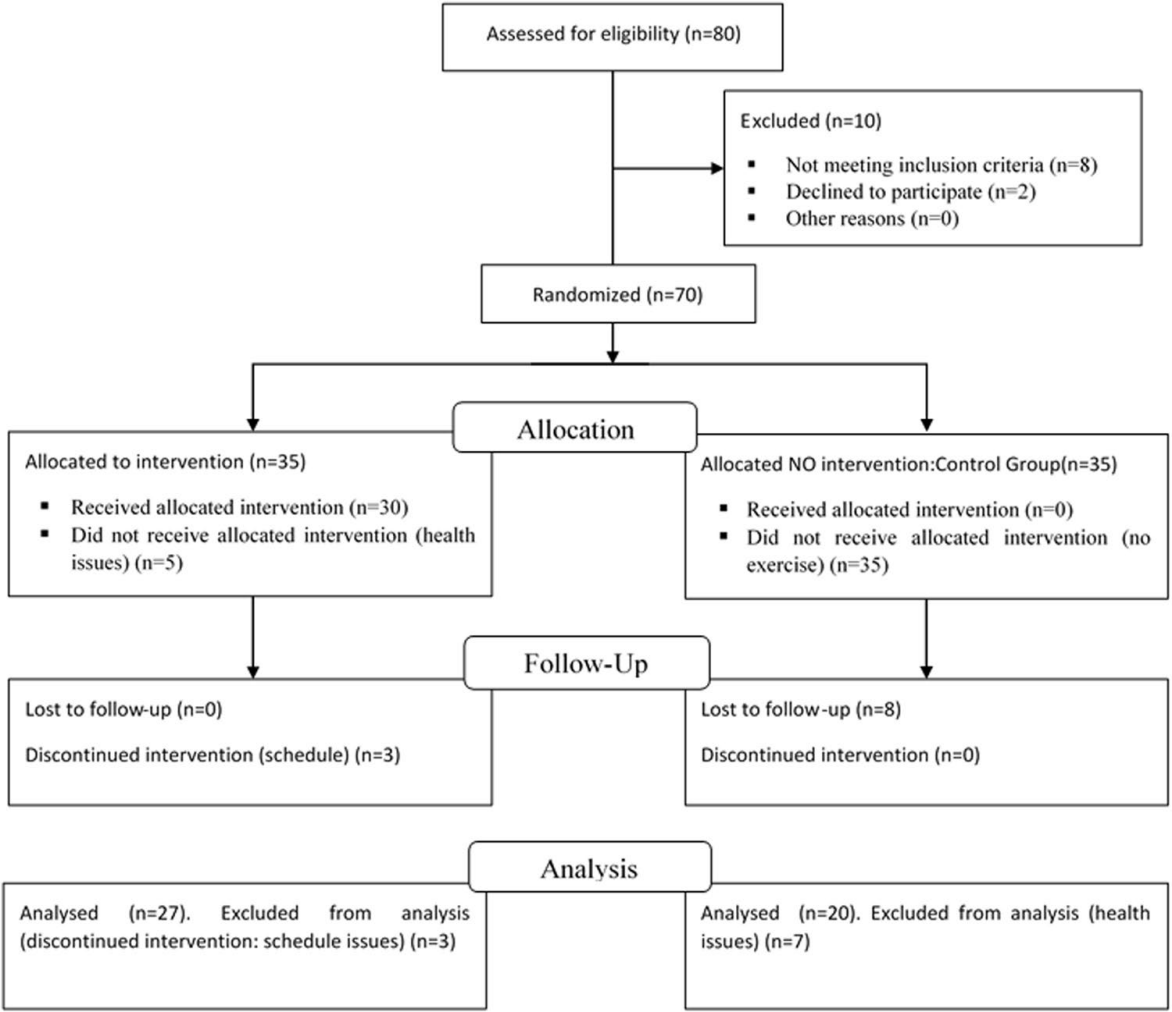
Flow diagram for sample configuration.

The study included a total of 47 subjects, (29 females, 18 males) aged 65 to 75 years (age: 68.7 ± 3.04 years; body height: 1.67 ± 0.09 meter; body weight: 69.59 ± 10.55 kg), recruited from a social club for the elderly. A simple randomized sampling was performed to group the volunteers into two groups: experimental group (n = 27; 10 men and 17 female) and control group (n = 20; 8 men and 12 female) (see Fig. 1). All subjects originated from Murcia (Spain) and met the following inclusion criteria: had never previously attended classes in fitness academies or were not currently performing regular physical activity, and had no previous experience with resistance-training programmes. Exclusion criteria included any history of neuromuscular, metabolic, hormonal or cardiovascular diseases; and taking any medication that could influence hormonal and neuromuscular metabolism. In addition, the participants were advised to not alter their diet during the study. Participants were informed about the possible risks and discomforts that could arise and were asked to complete a health history questionnaire and sign a consent form. The current study was approved of the Ethics Committee of the Catholic University San Antonio of Murcia (Spain) following the guidelines of the Helsinki Declaration.

### Design

A quasi-experimental design (2 × 2) was developed, dividing the participants into two groups: one experimental and one control. In this design, an initial measurement prior to the intervention (pre-test) and a measurement after the intervention (post-test) were conducted. This same research design has been used in previous studies that have sought to assess the effect of an intervention programme on body composition^59^, motivational variables^60,61^ and resistance-training^62,63^.

## Data Collection and Procedure

### Motivational variables

#### Behavioural regulation during exercise

The Spanish version^46^ of the Behavioural Regulation during Exercise Questionnaire-3^64^ was used. This questionnaire allows an evaluation of the type of motivational regulation related to physical exercise, and consists in 23 items distributed in three dimensions, based on the established distinction in the SDT between autonomous motivation, controlled motivation and amotivation. Autonomous motivation was calculated using the mean score of intrinsic regulation (e.g. “Because I think that exercise is fun”), integrated regulation (e.g. “Because I believe that physical exercise is consistent with my values”) and identified regulation (e.g. “I value the benefits of exercise”). Each of these regulations was composed of 4 items, except for the identified regulation that was composed of 3 items. Controlled motivation was calculated using the mean score of introjected regulation (e.g. “I feel guilty when I don’t exercise”) and external regulation (e.g. “I feel under pressure from my friends/family to exercise”) with 4 items each. Amotivation (e.g. “I don’t see why I should have to exercise”) was also calculated using 4 items. Previous research in the physical activity and health context demonstrated the internal reliability of the instrument^65^.

#### Basic psychological needs during exercise

To assess the satisfaction of the perceived needs of the participants, the Spanish adaptation of the Basic Psychological Needs in Exercise Scale was used (BPNES)^66^, which is specific to the context of physical activity and health^67^. The questionnaire includes 12 items distributed in three dimensions. Four items measure autonomy (e.g. “I exercise according to what I intend to do”), four items measure competence (e.g. “I feel that physical exercise is an activity that I do very well”) and the other four items measure relatedness (e.g. “I have a close relationship with the people with whom I exercise”). Previous research in the physical activity and health context demonstrated the internal reliability of the instrument^68^.

The answers to the questionnaires were assessed on a Likert scale ranging from 1 to 7, where one corresponded with the anchor statement “*strongly disagree*” and seven with the anchor statement “*strongly agree*”. Before carrying out the intervention, the main researcher informed the director of the social club of the purpose of the study. Likewise, all the participants involved were informed about the process that they were going to follow, emphasizing that participation was voluntary. The researcher overviewed how to complete the questionnaire and answered any questions that arose during the process. The different questionnaires were completed in a suitable environment to allow concentration, with each participant taking approximately 15–20 minutes to complete the questionnaires.

### Body composition variables

Total body weight was measured, after removal of shoes and heavy outer clothing, using a Tanita BC-418 MA scale, (Tanita Corporation, Arlington Heights, IL) to the nearest 0.1 kg^69^. Standing height without shoes was measured using a Seca 202 stadiometer (Seca, Hamburg, Germany) to the nearest 0.1 cm. To minimize the potential source of Bioimpedance systems (BIA) related to total body weight and height, body composition assessment was performed by a level two anthropometrist following International Society for the Advancement of Kinanthropometry recommendations^70^. Body Mass Index (BMI) was calculated as the ratio of weight to squared height. BMI was categorized into underweight (<18.50 kg/m^2^), normal weight (18.50–24.99 kg/m^2^), overweight (25.00–29.99 kg/m^2^), and obese (≥30.00 kg/m^2^)^71^.

Bioelectrical impedance was measured using the Tanita BC-418 MA scale, with participants standing barefooted on the analyser’s footpads, and holding its handles. Fat mass (FM) and muscle mass (MM) were then calculated, using the prediction equation described by Jebb *et al*.^72^. Bioelectrical impedance was chosen due to its ease of use and less invasive nature, making it suitable for assessment of body composition in vulnerable populations such as obese individuals and the elderly^69^.

### Motivational resistance-training intervention programme

Figure 1 presents the flow diagram of the intervention. Before starting the intervention, it was necessary to conduct a period of training with the instructor. The first author led the training process that lasted three weeks. During the first week, the instructor read four papers about SDT and health. These documents orientated the instructor to the importance of the motivation of the elderly in the physical activity context. Once the instructor had read this material, the first author conducted an individual meeting with the instructor to discuss the content of the papers. In this meeting, the first author and instructor began discussions about planning an intervention using the different motivational strategies. In week two, the first author and instructor designed the sessions that were subsequently applied to the participants. In this phase, objectives, task and motivational strategies were established for each session. In week three, the instructor carried out three sessions with three different groups of elderly people who did not participate in the actual study. After each session, which were observed by the first author, a post-lesson reflection meeting was held to discuss strengths and areas in which instructor and first author felt the sessions could be improved. During these reflection meetings, the first author linked discussions to the motivational strategies that were designed for application in the current study.

**Figure 2:**
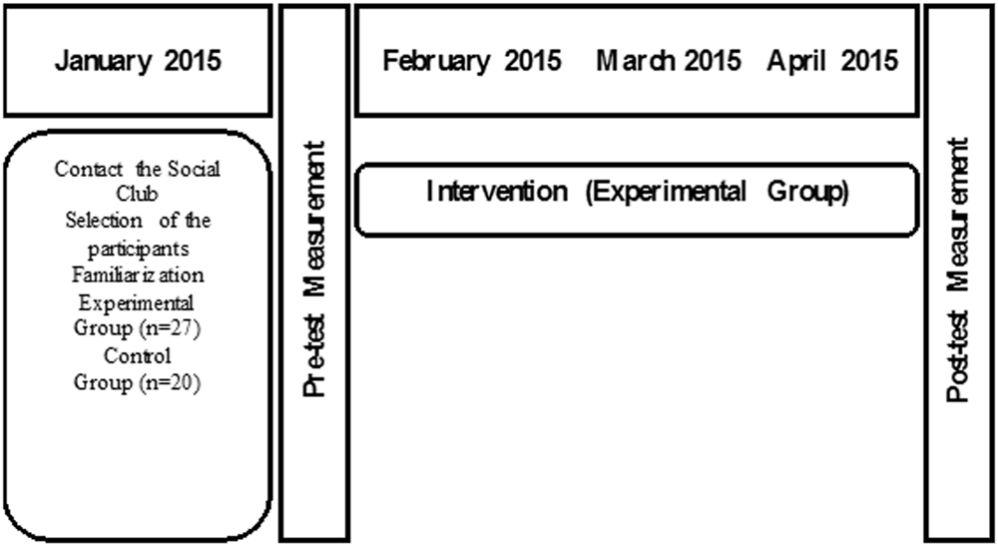
Timeline of the study.

Once the teacher training process was completed, the subjects were submitted to four weeks of training, two sessions per week, in order to be familiarized with the resistance-training exercises performed in the current study. During this familiarization period, a higher emphasis was placed on learning the proper exercise techniques, and brief pauses between repetitions were allowed in order to reset their starting positions when necessary^73^. During the second week of the familiarization period, a pre-test of the motivational variables was performed, as well as measuring weight, height, BMI, FM and MM. Both pre-test and post-test were carried out in one day between 8:00 and 9:00 a.m. After the pre-test, the experimental group underwent a resistance-training programme for 12 weeks, where different motivational strategies were used. The resistance-training programme incorporated resistance exercises of six major regions and consisted in 3 training sessions per week on non-consecutive days (Monday, Wednesday and Friday). The six regions were chest, back, triceps, biceps, shoulders and legs. The different regions were grouped into a circuit. The control group, on the other hand, did not participate in the resistance-training programme Fig. 2.

The experimental group exercised at a moderate intensity (8 to 12 repetitions). The load was increased during the 12 weeks from 65% 1-RM to 80% 1-RM. This training load was increased when the individual could perform more than the prescribed number of repetitions (12 repetitions), following the OMNI-RES scale and a hard effort perception level^74^. A 1–2 minutes resting period was allowed between sets. During the sessions, the participants were verbally encouraged to perform the complete range of motion for each exercise. There was no attempt to control the velocity of the repetitions performed. Prior to each training session, the volunteers performed a specific warmup, consisting of 10 repetitions with approximately 50% of the load used in the first and second exercises of the training session. A total of 36 sessions were performed during the training period. The instructor monitored the attendance of the participants to the sessions and adherence to the resistance-training programme was 100% for all participants. All training sessions were monitored by an experienced physical education instructor and the subjects were not allowed to perform any additional exercises during the training period. During the training weeks, participants were instructed not to change dietary habits or perform additional non-resistance-training related physical activity.

In order for the resistance-training programme to be motivating and increase adherence, a series of motivational strategies were performed. In this sense, motivation is considered important for successful changes in physical activity behaviour^75^. The strategies used were based on the principles of the Self-Determination Theory (SDT)^38,40,49^. SDT includes a meta-theory for framing motivational studies, a formal theory that defines intrinsic and varied extrinsic sources of motivation, their role in cognitive and social development, as well as in individual differences^38–40,76^. SDT is a theory of motivation and personality that addresses three universal, innate and basic psychological needs: autonomy, competence and relatedness. In this sense, and in order to increase the intrinsic motivation in the sessions, it is necessary that instructors propose tasks in which the elderly feel competent, relate to others and have the possibility to make decisions and act on their own initiative^47^. During the resistance-training programme, the subjects received verbal motivational strategies, based on previous studies which have proven the beneficial effect they exert on BPN satisfaction and autonomous motivation^28,47,77–79^. Based on those previous studies, the strategies adapted and designed for the programme were:

#### Strategies based on autonomy

1. *Educate elderly practitioners about the benefit of a resistance-training programme*: The first step is to inform of the benefits of exercise and the need to increase the level of physical activity for long-term weight control. For example, resistance-training may help preserve fat-free mass during weight loss; resistance-training increases energy expenditure. The amount of energy expenditure depends on the intensity, duration of the activity, and on the muscle group involvement. The increase in energy consumption associated with activity occurs primarily during the activity itself, but there is also a short-lived post exercise increase in metabolic rate. In addition, weight maintenance can be achieved with either programmed or lifestyle activity. Increasing daily lifestyle activities is as effective as a structured exercise programme in maintaining long-term weight loss.

2. *Explain the purpose of the resistance-training programme*: In this manner, the positive perception of the activity is increased, as well as the participant’s commitment and sense of autonomy. The goals of the resistance-training session and specific objectives are explained (for example, what muscle groups are involved in each exercise).

#### Strategies based on competence

3. *Encourage the perception of competence by the participant*: Have the participants focus on improving their own task, avoiding external pressures that may cause stress (e.g. the instructor orients the participants to only focus on improving their own tasks, while providing positive feedback such as “good job, you’re doing fine”).

4. *Establish moderately difficult objectives*: Emphasize the importance of progression and setting realistic goals. This strategy can help extend the practice time, and serve as a stimulus to initiate and/or maintain the intentions of being physically active (e.g. teach the participants to progress towards the perception of effort and increased loads; it is necessary to explain to the participants that their body will need a number of adjustments before the effects of hypertrophy may be visible. To this end, it is necessary to perform muscle-strengthening exercise and progressively increase the load and repetitions).

5. *Take into account the information provided by the elderly practitioner during resistance-training programme*: This information will be obtained during and after the sessions, by the participants sharing their views with the instructor who takes them into account for future interactions (e.g. the instructor must monitor the feelings of the practitioner and know their evolution with training loads, to properly perform the tasks and avoid injury).

6. *Convey an adequate task environment*: Effort and personal improvement should be prioritized, avoiding social comparison. In this manner, it is more likely for the participants to continue to attend the sessions by emphasizing the positive results (for example, explaining to the participant how he/she has improved in the execution of an exercise with free weights, and how with practice will perform the technique more efficiently and increase the load). Also, it is recommended to comment on the values of the task being performed, by applying psychological training techniques such as self-instruction, mentally performing the task, and relaxation. These aspects correlate with a positive task environment, making the participant aware of the benefits of physical activity for physical, psychological and social health.

7. *Encourage the participants by emphasizing that the activity can be improved through practice*: This consists in redirecting negative emotions (i.e. I cannot do this; I have no resistance; I cannot increase the load) towards positive ones (If you practise long enough you will improve; everyone has their own pace, no hurry; if you practise you will see that it is not that difficult; if you do not try you will not know what you are capable of, etc.).

*8. Offer clear feedbacks*: The instructors should inform the participants of the exercises they have performed properly, as well as the mistakes they made. The instructors must always have a positive attitude of encouragement, focusing on how the participant can improve through practice (e.g. recognize the individual’s progress and improvement, ensure equal opportunities to all the participants, focus on self-development, congratulate them when improving).

#### Strategies based on social relations and enjoyment

9*. Encourage the relationship between participants*: One of the most feared aspects of aging is loneliness. To this end, the training sessions are focused to encourage social relations, which are separated into two phases in order to enhance group cohesion and involvement in the activities. The first phase involves the activity itself (task cohesion), where the instructors may encourage a participant who is resting to motivate a partner, or have the participants make joint decisions. The second phase occurs after the session (social cohesion) by encouraging other activities (e.g. proposing mini-events, go together for a healthy breakfast after the training session, attend sports events, etc.).

10. *Have the participants enjoy the activities*: The participants will be more motivated if they really enjoy what they are doing. The instructors must have previous knowledge of the group members, their concerns and requirements, in order to achieve the objectives while enjoying the activities (for example, use music to motivate the participants and perform enjoyable resistance-training activities).

In summary, these 10 strategies, based on previously published studies, increase adherence and enjoyment of the resistance-training programme. In this manner, the participants are less likely to abandon the training programme due to lack of motivation, boredom, or lack of knowledge of the benefits resistance exercise has on their health.

### Data analysis

The statistical program SPSS v21.0 (Chicago, IL) was used for the data analysis and processing. Preliminary analyses included the testing of assumptions such as normality, homogeneity of variances and multicollinearity. No violations in data normality were evident from the Shapiro-Wilks test, which led to the use of parametric statistics. However, examination of the pre-test data through a multivariate analysis of variance (MANOVA) test to examine if there were statistically significant differences in the dependent variables revealed significant differences between the two groups in some of the dependent variables. Pre-test scores were therefore included as covariates in subsequent analysis, both in motivational variables and in body composition variables. No violations of assumptions in terms of multicollinearity were noted after examining the correlations between dependent variables both in the pre-test and post-test measures, with no Spearman values over 0.70.

In the motivational variables, Cronbach’s alpha coefficient was employed to calculate the internal reliability for each questionnaire item. In these types of variables, to compare the mean scores of each group while controlling for pre-test differences, a MANCOVA was conducted to examine if there were statistically significant differences in this type of variable between the two groups. Pairwise comparisons were analysed (with Bonferonni correction) when a significant overall effect was detected.

Likewise, the body composition variables were analysed according to MANCOVA to examine if there were statistically significant differences in this type of variable between the two groups. On the pre-test scores there were no statistically significant differences at the multivariate level, but in this case, the variable body mass index was included as covariable^80^. Pairwise comparisons were analysed (with Bonferonni correction) when a significant overall effect was detected. Data corresponding to body compartments were represented as percentages or kg. The percentage of body FM at the beginning of the study (%FM0) was compared with the percentage of body FM at the end of the study (%FMt). The difference between both values (Δ%FM = %FMt − %FM0) indicated the variation in fat component of each participant during the study. The same rationale was used for changes in the body muscle mass in kg (ΔkgMM = kgMMt − kgMM0), kgMM0 and kgMMt being the amount of body MM at the beginning and end of the study, respectively. The changes in body weight (BW) expressed in kg were calculated by the difference between the weight at the beginning and end of the study (ΔBW = BWt − BW0). For BMI changes, expressed in kg/m^2^, these were calculated by the difference between the BMI at the beginning and end of the study (ΔBMI = BMIt − BMI0). Positive values in ΔBW, ΔBMI, Δ%FM and ΔkgMM are indicative of an increase in the corresponding indicator, while negative values indicate a decrease^81^.

For both types of variables, the level of statistical significance was established at *p* ≤ 0.05, with a confidence interval for differences of 95%. Effect sizes were calculated using the partial eta-squared statistic (ηp2) to establish the substantive meaningfulness of the differences found.

## Results

### Preliminary analysis

The results of the initial MANOVA for motivational variables on the pre-test scores indicated that there were no statistically significant differences at the multivariate level (Pillai’s Trace *V* = 0.132; *F*(6, 40) = 1.014; *p* = 0.430; η*p*^2^ = 0.132; *SP* = 0.352). Despite the results at the multivariate level, in the pairwise comparisons, there were statistically significant differences between groups in competence (F = 4.088; *p* = 0.049; η*p*^2^ = 0.083). There were no statistically significant differences for autonomy (F = 0.665; *p* = 0.419; η*p*^2^ = 0.015), relatedness (F = 1.270; *p* = 0.266; η*p*^2^ = 0.027), autonomous motivation (F = 2.959; *p* = 0.092; η*p*^2^ = 0.062), controlled motivation (F = 0.503; *p* = 0.482; η*p*^2^ = 0.011) and amotivation (F = 1.704; *p* = 0.198; η*p*^2^ = 0.036). On the other hand, in body composition variables there were no statistically significant differences at the multivariate level (Pillai’s Trace *V* = 0.083; *F*(4, 42) = 0.951; *p* = 0.444; η*p*^2^ = 0.083; *SP* = 0.275). In the pairwise comparisons, there were no statistically significant differences for body weight (F = 0.073; *p* = 0.788; η*p*^2^ = 0.002), body mass index (F = 1.512; *p* = 0.225; η*p*^2^ = 0.033), muscle mass (F = 0.723; *p* = 0.400; η*p*^2^ = 0.016) and fat mass (F = 1.801; *p* = 0.186; η*p*^2^ = 0.038).

### Motivational variables

The internal consistency coefficient (Cronbach’s Alpha) of the motivational variables and descriptive statistics of the variables are shown in Table 1. All the motivational variables indicated an acceptable reliability, exceeding the 0.70 criterion^82^.

**Table 1:** Descriptive statistics and inter-group analysis for motivational variable.

Variables	**α**	Test-Time	Experimental Group (*n* = 27)		Control Group (*n* = 20)		Mean Difference	*F*(gl)	*p*	*η*_p_^2^	OP	95% CI
			*M*	*SD*	*M*	*SD*						
Autonomy	0.76	Pre	4.20	0.720	4.01	0.886	0.191	0.665	0.419	0.015	0.126	[−0.281, 0.664]
		Post	4.32	0.689	3.76	0.699	0.562	7.527	**0.004**	0.197	0.854	[0.138, 0.660]
Competence	0.75	Pre	4.20	0.596	3.77	0.858	0.429	0.665	0.**049**	0.083	0.508	[0.002, 0.856]
		Post	4.37	0.577	3.51	0.671	0.858	22.083	**<0.001**	0.438	0.998	[0.346, 0.746]
Relatedness	0.75	Pre	4.45	0.509	4.20	1.01	0.254	1.27	0.226	0.027	0.197	[−0.200, 0.707]
		Post	4.60	0.434	3.90	0.856	0.702	13.524	**<0.001**	0.304	0.981	[0.243, 0.710]
Autonomous Motivation	0.78	Pre	4.25	0.610	3.85	0.991	0.403	2.959	0.092	0.623	0.391	[−0.069, 875]
		Post	4.15	0.642	3.63	0.772	0.522	6.376	0.137	0.056	0.316	[−0.058, 0.403]
Controlled Motivation	0.80	Pre	2.96	0.553	3.63	0.772	0.194	0.503	0.482	0.011	0.107	[−0.254, 0.530]
		Post	3.05	0.659	2.75	0.641	0.304	2.49	0.301	0.027	0.176	[−0.125, 0.396]
Amotivation	0.82	Pre	1.51	0.716	1.81	0.822	0.294	1.70	0.198	0.036	0.248	[−0.748, 0.160]
		Post	1.72	0.763	1.85	0.848	−0.128	0.293	0.664	0.005	0.071	[−0.239, 0.372]

M = Mean; SD = Standard Deviation; **α** = Cronbach’s alpha.

The MANCOVA indicated a significant effect between the experimental and control groups in the post-test measurement (Pillai’s Trace *V* = 0.638; *F*(6, 34) = 10.004; *p* < 0.001; η*p*^2^ = 0.638; *SP* = 0.998). Specifically, significant differences were detected between the two groups (in favour of the experimental group) in the following variables: autonomy (F = 9.549; *p* = 0.004, η*p*^2^ = 0.197); competence (F = 30.397; *p* < 0.001, η*p*^2^ = 0.438); relatedness (F = 17.068; *p* = 0.001, η*p*^2^ = 0.304). No significant differences were observed between the two groups in the following variables: autonomous motivation (F = 2.301; *p* = 0.137, η*p*^2^ = 0.056); controlled motivation (F = 1.100; *p* = 0.301, η*p*^2^ = 0.027) and amotivation (F = 0.192; *p* = 0.664, η*p*^2^ = 0.005) (Table 1).

### Body composition variables

The MANCOVA indicated a significant effect in the group variable (Pillai’s Trace *V* = 0.479; *F*(4, 38) = 8.741; *p* < 0.001; η*p*^2^ = 0.479; *SP* = 0.998). The experimental group significantly decreased their FM percentage and increased MM (in kg) with respect to the control group. Furthermore, significant differences were observed regarding BW and BMI values. In this case, the control group presented increased BW and BMI values after the intervention, while these values decreased in the experimental group (Table 2).

**Table 2:** Descriptive statistics and inter-group analysis for body composition variables.

	Experimental Group (*n* = 20)		Control Group (*n* = 27)		Mean Difference	*F*(gl)	*p*	η_*p*_^2^	OP	95% CI
	*M*	*SD*	*M*	*SD*						
ΔBody weight (kg)	0.263	1.28	1.140	0.57	−0.854	7.33(1, 44)	**0.010**	0.143	0.754	[−1.490, 0.218]
ΔBMI (kg/m^2^)	0.110	0.49	0.410	0.21	−1.376	7.36(1, 44)	**0.020**	0.116	0.653	[−0.535, −0.047]
ΔMM (kg)	1.274	1.11	0.290	1.23	1.056	9.23(1, 44)	**0.004**	0.174	0.844	[−1.756, −0.356]
ΔFM (%)	−1.407	1.64	−0.150	1.77	−0.291	5.785(1, 44)	**0.009**	0.143	0.756	[−2.398, −0.354]

M = Mean; SD = Standard Deviation; BMI = Body Mass Index; MM = Muscle Mass; FM = Fat Mass.

Δ = difference between the variable at the beginning and end of the study.

## Discussion

The main objective of the study was to analyse the effect of a motivational resistance-training programme in the aging population. The programme included motivational strategies in order to satisfy the basic psychological needs of the participants, as well as self-determining motivation and body composition. The aging process causes structural and functional changes that may progressively affect the individual’s health. Therefore, a regular exercise routine is an efficient method to maintain a healthy wellbeing in this population^1,6,83^. Despite this, few individuals over 65 years of age include regular physical exercise as part of their lifestyle. This has resulted in recent years in researchers developing intervention strategies that encourage physical activity, with special attention to the social aspects, in order to increase their adherence to physical exercise and make these individuals more physically active^84^.

Our first hypothesis in this study was that the motivational strategies included in the resistance-training programme may give the experimental group a higher level of satisfaction of their basic psychological needs, and consequently, a more self-determining motivation and adherence. Indeed, the results indicated that both BPN and autonomous motivation increased in the experimental group compared to the control, confirming our first hypothesis. The results observed here with respect to the BPN coincide with previous studies, where special attention was given to the intervention method used, by creating an environment that was focused on autonomy and social relations with the participants^38,42,75,76,85^. In this sense, the majority of the studies confirm that a higher level of adherence to physical exercise correlated with an increase in BPN satisfaction^45^. Therefore, to encourage the elderly to adopt a more active lifestyle, sports instructors must design the sessions considering the specific needs of each participant, with several levels of difficulty, as well as encourage group and cooperative activities^47,86^.

On the other hand, many studies have shown a strong correlation between BPN and self-determining motivation, since they are essential for psychological growth and integration, as well as for social and constructive development and personal wellbeing^38,41,42^. In this manner, an individual will possess more autonomous motivation when he/she is more efficient in performing a certain task, as well as when the individual perceives a higher sense of choice and is better regarded by other relevant individuals. All this, in turn, will give rise to more positive consequences (e.g. enjoyment, self-esteem), which will ultimately increase their adherence to the training programme for at least the duration of the intervention^75,87,88^. Along this line, and based on the obtained results as well as previous studies, the instructors must fully understand the main objectives of the resistance-training programme, that is, to adapt the exercise to the level of the participants so that they may perceive them as moderately difficult in order to encourage improvement, while maintaining a motivational environment. Also, the instructors must allow for group efforts, in order to allow social interactions among the participants. Overall, this approach will improve the adherence to physical exercise and consequently the adoption of a more active lifestyle^89–91^.

The second hypothesis corresponds to how motivational resistance-training can influence body composition, decreasing fat mass and BMI while increasing muscle mass. The results of this study confirm the hypothesis, as the experimental group significantly improved their body composition with respect to the control group. In addition, previous studies corroborate the results, indicating that resistance-training programmes are capable of improving body composition in the aging population^11,92–96^. In this vein, a previous study by Villanueva *et al*.^95^. based on a resistance-training intervention, obtained significant improvements in body composition, both when compared with themselves before and after the intervention as well as when compared with the control group. A similar result was observed by Straight *et al*.^96^, where the experimental group significantly reduced their fat mass and BMI after the training programme, compared to the control group.

The motivational resistance-training programme used in the present study gave positive results after only 12 weeks of intervention. In this way, the results indicated that the intervention carried out improved the body composition in the aging population and indirectly muscle function, lipid profile, glycaemia, etc.^97–101^. As our study indicated, evidence was accrued for the relevance of need supportive consultations to achieve corresponding changes in participants’ basic need satisfaction and motivation for engagement. These motivational processes were predictive of participants’ emotional wellbeing and levels of moderate-vigorous physical activity post-programme^102,103^. Despite the fact that there is an increased interest in research regarding the benefits of resistance-training in body composition, muscle mass, strength, sarcopenia and osteoporosis, among others, few investigations have focused on analysing the effects of a training programme on physical and psychosocial factors in the elderly. It should be noted that in this population, resistance-training programme design should not only be oriented towards physical variables, but should also emphasize the improvement of social variables that may act as predictors of the intention to be physically active^47,104^. Therefore, the present study suggests that instructors and coaches specifically design motivational resistance-training programmes for the elderly that include motivational strategies to encourage adherence, with the objective of inducing physical, psychological and social improvements, which are what the World Health Organization defines as a healthy lifestyle.

Although the results may indicate the positive effect of a motivational resistance-training programme on motivational variables and body composition variables, the results should be taken with caution because this is a preliminary study with a small sample, which limits the capacity to extrapolate the results. This is due to the difficulty in obtaining a large number of elderly volunteers, as the majority do not contemplate including regular exercise as part of their routine. In fact, many declined when informed that they were to undergo a resistance-training programme, possibly due to fear or lack of knowledge of the benefits. With a larger number of participants, it would have been possible to analyse the benefits of performing the intervention for more than 12 weeks, as well as to discern if better results would have been obtained with 3 or 4 sessions/week. Consequently, future research can extend the current sample by, for example, increasing the number of participants from different social clubs for the elderly and different locations in the same region. Also, the analysis of more motivational variables would have been interesting, especially those related to the participants’ perception of the motivational environment proposed by the instructor, in order to improve the adherence to the resistance-training programme. Finally, combining the training programme with diet and nutrition adapted to the energy requirements of the participants would possibly further enhance the effects observed in the elderly participants.
